# Quantitative cross-linking via engineered cysteines to study inter-domain interactions in bacterial collagenases

**DOI:** 10.1016/j.xpro.2023.102519

**Published:** 2023-08-20

**Authors:** Jamil Serwanja, Hans Brandstetter, Esther Schönauer

**Affiliations:** 1 Department of Biosciences and Medical Biology, Paris Lodron University of Salzburg, Salzburg 5020, Austria

**Keywords:** Molecular Biology, Protein Biochemistry, Structural Biology

## Abstract

Inter-domain movements act as important activity modulators in multi-domain proteins. Here, we present a protocol for inter-domain cross-linking via engineered cysteines. Using collagenase G (ColG) from *Hathewaya histolytica* as a model, we describe steps for the design, expression, purification, and cross-linking of the target protein. We detail a system to monitor the progress of the cross-linking reaction and to confirm the structural integrity of the purified cross-linked proteins. We anticipate this protocol to be readily adaptable to other multi-domain enzymes.

For complete details on the use and execution of this protocol, please refer to Serwanja et al.^1^

## Highlights

• Inter-domain cross-linking for reversible conformational locking of multi-domain proteins
• Production of monodisperse, homogenously cross-linked, folded proteins
• Orthogonal validation of structural integrity of cross-linked proteins

## Abstract (editor highlights)

Publisher’s note: Undertaking any experimental protocol requires adherence to local institutional guidelines for laboratory safety and ethics.

## Graphical abstract

**Figure:**
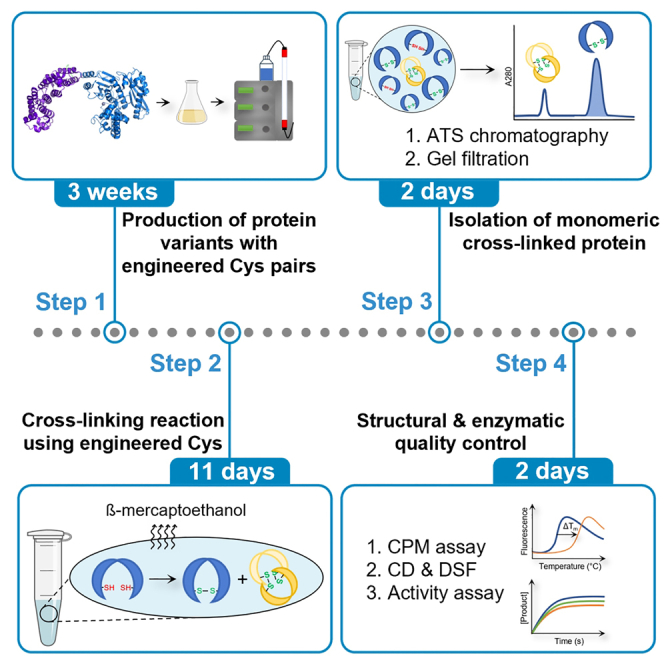

## Before you begin

Inter-domain movements act as important activity modulators in multi-domain proteins; whether it is by creating binding surfaces for ligands,^2^^,^^3^^,^^4^ or by enabling catalysis or receptor activation.^3^^,^^5^^,^^6^^,^^7^ To unravel their functional significance, it is crucial to be able to lock the target domains in distinct conformational states. This can be done by inter-domain cross-linking via engineered cysteine residues.^8^^,^^9^^,^^10^ This method has the advantage that the target protein can be reversibly maintained in a fixed conformational state. Upon addition of a reducing agent, the restraining linkage dissolves. Yet, this method entails pitfalls that need to be carefully handled, as one must avoid the presence of i) mislinked higher oligomeric species, and ii) a mixture of cross-linked and non-cross-linked proteins. Moreover, it is also vital that the engineered cysteine pair and the subsequently formed disulfide bond do not compromise the fold of the protein or annihilate critical functional residues or motifs.

Here we describe the cross-linking of the collagenase unit (CU) of the collagenase ColG from *Hathewaya histolytica*. The CU is composed of the activator domain (AD) and peptidase domain (PD) and adopts an open conformation in the crystal structure. A two-state model of collagen degradation was proposed, in which the CU switches between an open and closed conformation.^11^ This protocol was established to generate cross-linked CU variants in different states of closure. Care was taken to address the aforementioned pitfalls, in order to produce pure, functional, homogenously cross-linked, monomeric protein.

This protocol can be easily adapted for other multi-domain proteins, but it is important to consider the following points beforehand:

1. In case the target protein contains conserved, functionally relevant cysteines (e.g., cysteine proteases, disulfide bonds), this protocol will require adaptations or may not even be applicable, as mutation of these residues and/or the introduction of additional cysteines will interfere with proper folding and protein activity.
2. When designing the cross-linked protein variants, please follow these guidelines:
  a. Perform a multiple sequence alignment of the target protein with known homologues to identify conserved residues.
  b. Do not target conserved amino acids for mutation, as sequence conservation is indicative for residues of structural and/or functional significance.
  c. Remove naturally occurring non-conserved cysteine residues by mutation; otherwise the yield of properly cross-linked protein will be severely reduced.
  d. Visualize the conformational state that you want to stabilize using software programs such as PyMol^12^ or UCSF Chimera^13^:
    i. Use available crystal structures of the target protein, homology models or AlphaFold^14^ models.
    ii. Model the targeted conformational state of the two involved domains.
    iii. Search for surface-accessible sidechains facing each other at the interface of the two domains (C_β_–C_β_ distance <5 Å). Upon mutation into cysteines, this will translate into a sulfur-sulfur distance of approx. 2 Å, ideal for disulfide-bond formation, if the hypothetical conformation is populated.^15^ If two residues for exchange cannot be identified, more distant pairs may be used together with bridging cross-linkers.

## Key resources table

**Table:** 

REAGENT or RESOURCE	SOURCE	IDENTIFIER
**Chemicals, peptides, and recombinant proteins**		

Ni-NTA Superflow	QIAGEN GmbH	30430
β-mercaptoethanol (ßME)	Sigma	M3148-100ML
Superdex 200 10/300 GL SEC column	Cytiva	GE28-9909-44
Activated Thiol Sepharose 4B	GE HealthCare	T8512-5G
Dithio-bismaleimidoethane cross-linker (DTME)	Thermo Scientific	PIER22335
7-Diethylamino-3-(4-maleinimidophenyl)-4-methyl coumarin (CPM)	Sigma	96669-10MG-F
Mca-Ala-Gly-Pro-Pro-Gly-Pro-Dpa-Gly-Arg-NH_2_ (quenched-fluorescent peptidic substrate)	Schönauer et al.^16^	

*Clostridium histolyticum* collagenase unit (ColG-CU)	Eckhard et al.^11^	Uniprot: Q9X721

**Other**		

Infinite M200 plate reader	TECAN	NA
PCR thermocycler	Eppendorf	NA
Horizontal tube mixer	Ingenieurbüro CAT, M. Zipperer GmbH	NA
AKTA FPLC purifier	GE HealthCare	NA
Corning 3686 Black half area 96 well plates	Corning Incorporated	Corning 3686
Magnetic stirrer	NA	NA
Tycho NT.6 instrument	NanoTemper Technologies	
Tycho NT.6 Capillaries	NanoTemper Technologies	Cat# TY-C001
SORVALL Evolution RC centrifuge	SORVALL Evolution RC	NA
Bench laboratory centrifuge	Sigma	4K15C
Amicon Ultra-15 centrifugal filter units	Millipore	NA
Chirascan Plus CD spectrophotometer	Applied Photophysics	NA
Circular dichroism cuvette, UV quartz, bottleneck, light path 0.5 mm	Starna Scientific	NA
NANODROP 2000C spectrophotometer	Thermo Scientific	NA

**CRITICAL:** 7-diethylamino-3-(4-maleinimidophenyl)-4-methyl coumarin (CPM) is light sensitive. Dissolve in DMSO to a final concentration of 4 mg/mL and store 50 μL aliquots at −80°C in an opaque storage container.

## Materials and equipment

**Table:** Loading buffer / Wash buffer 1

Reagent	Final concentration	Amount
NaH_2_PO_4_ pH 8.0 (0.5 M)	50 mM	100 mL
NaCl (5.0 M)	300 mM	60 mL
Imidazole (4.0 M)	10 mM	2.5 mL
β-mercaptoethanol (14.3 M)	10 mM	0.7 mL
dH_2_O	N/A	836.8 mL
**Total**	**N/A**	**1,000 mL**

Store at 4°C for up to 2 months.

**Table:** Wash buffer 2

Reagent	Final concentration	Amount
NaH_2_PO_4_ pH 8.0 (0.5 M)	50 mM	100 mL
NaCl (5.0 M)	1000 mM	200 mL
Imidazole (4.0 M)	10 mM	2.5 mL
β-mercaptoethanol (14.3 M)	10 mM	0.7 mL
dH_2_O	N/A	696.8 mL
**Total**	**N/A**	**1,000 mL**

Store at 4°C for up to 2 months.

**Table:** Wash buffer 3

Reagent	Final concentration	Amount
NaH_2_PO_4_ pH 8.0 (0.5 M)	50 mM	100 mL
NaCl (5.0 M)	300 mM	60 mL
Imidazole pH 8.0 (4.0 M)	20 mM	5 mL
β-mercaptoethanol (14.3 M)	10 mM	0.7 mL
dH_2_O	**N/A**	834.3 mL
**Total**	**N/A**	**1,000 mL**

Store at 4°C for up to 2 months.

**CRITICAL:** Adjust to pH 8.0 at 20°C–22°C (hereafter denoted as RT).

**Table:** Elution buffer

Reagent	Final concentration	Amount
NaH_2_PO_4_ pH 8.0 (0.5 M)	50 mM	100 mL
NaCl (5.0 M)	300 mM	60 mL
Imidazole (4.0 M)	250 mM	62.5 mL
β-mercaptoethanol (14.3 M)	10 mM	0.7 mL
dH_2_O	N/A	776.8 mL
**Total**	**N/A**	**1,000 mL**

Store at 4°C for up to 2 months.

**CRITICAL:** Adjust to pH 8.0 at RT.

**Table:** Size exclusion chromatography buffer

Reagent	Final concentration	Amount
HEPES (1.0 M)	10 mM	10 mL
NaCl (5.0 M)	100 mM	20 mL
β-mercaptoethanol (14.3 M)	10 mM	0.7 mL
Glycerol (87%)	5%	57.5 mL
NaN_3_ (3.0 M)	3 mM	1 mL
ddH_2_O	N/A	910.8 mL
**Total**	**N/A**	**1,000 mL**

Store at 4°C for up to 2 months.

**CRITICAL:** Adjust to pH 7.5 at RT. Filter and degas immediately prior to usage.

**Table:** Oxidation buffer

Reagent	Final concentration	Amount
Tris-HCL, pH 8.5 (1.0 M)	50 mM	50 mL
NaCl (5.0 M)	300 mM	60 mL
Glycerol (87%)	5%	57.5 mL
NaN_3_ (3.0 M)	3 mM	1 mL
dH_2_O	N/A	831.5 mL
**Total**	**N/A**	**1,000 mL**

Store at RT for up to 1 month.

**CRITICAL:** Degas immediately prior to usage.

**Table:** Activated Thiol Sepharose regeneration solution

Reagent	Final concentration	Amount
2,2′-dipyridyl disulfide	1.5 mM (approximately 1.0 mg/mL)	250 mg
ddH_2_O	N/A	250 mL
**Total**	**N/A**	**250 mL**

Store at 4°C for up to 2 weeks.

**CRITICAL:** 2,2′-dipyridyl disulfide takes several hours to dissolve at RT.

Adjust to pH 8.0 and filter and degas buffer prior to use.

The use of gloves is crucial to avoid contamination of buffers and solutions with thiol-containing substances.

**Table:** Cleaning solution for Activated Thiol Sepharose column

Reagent	Final concentration	Amount
Triton X-100 (100%^v^/_v_)	0.1%^v^/_v_	1 mL
dH_2_O	N/A	999 mL
**Total**	**N/A**	**1,000 mL**

Store at RT for up to 3 months.

**Table:** CD spectroscopy buffer

Reagent	Final concentration	Amount
Tris-SO_4_, pH 7.5 (1.0 M)	15 mM	0.75 mL
NaF (1.0 M)	100 mM	5 mL
CaCl_2_ (1.0 M)	1 mM	0.05 mL
ddH_2_O	N/A	44.2 mL
**Total**	**N/A**	**50 mL**

Store at 4°C for up to 2 weeks.

**CRITICAL:** Adjust to pH 7.5 at RT.

**Table:** Buffer B1 (Peptidolytic assay)

Reagent	Final concentration	Amount
HEPES pH 7.5 (1.0 M)	250 mM	12.5 mL
NaCl (5.0 M)	400 mM	4 mL
NaN_3_ (3.0 M)	3 mM	0.05 mL
CaCl_2_ (1.0 M)	10 mM	0.5 mL
ZnCl_2_ (1.0 mM)	10 μM	0.5 mL
DMSO (100%^v^/_v_)	2.0%^v^/_v_	1 mL
ddH_2_O	N/A	31.45 mL
**Total**	**N/A**	**50 mL**

Store at 4°C for up to 1 month.

## Step-by-step method details

### Production of non-cross-linked protein

**Timing: 3 weeks**

This part of the protocol details the design, cloning, overexpression, and pre-purification of the engineered CU variants (CL1, CL2, CL3 CL4) in a non-cross-linked, i.e., reduced, state. The expression and purification steps are based on Hoppe et al*.*^17^

***Note:*** The individual cloning steps are not presented in detail here, as different cloning methods can be used to generate the necessary construct plasmids. Therefore, we only list the necessary cloning products.

**Figure 1:**
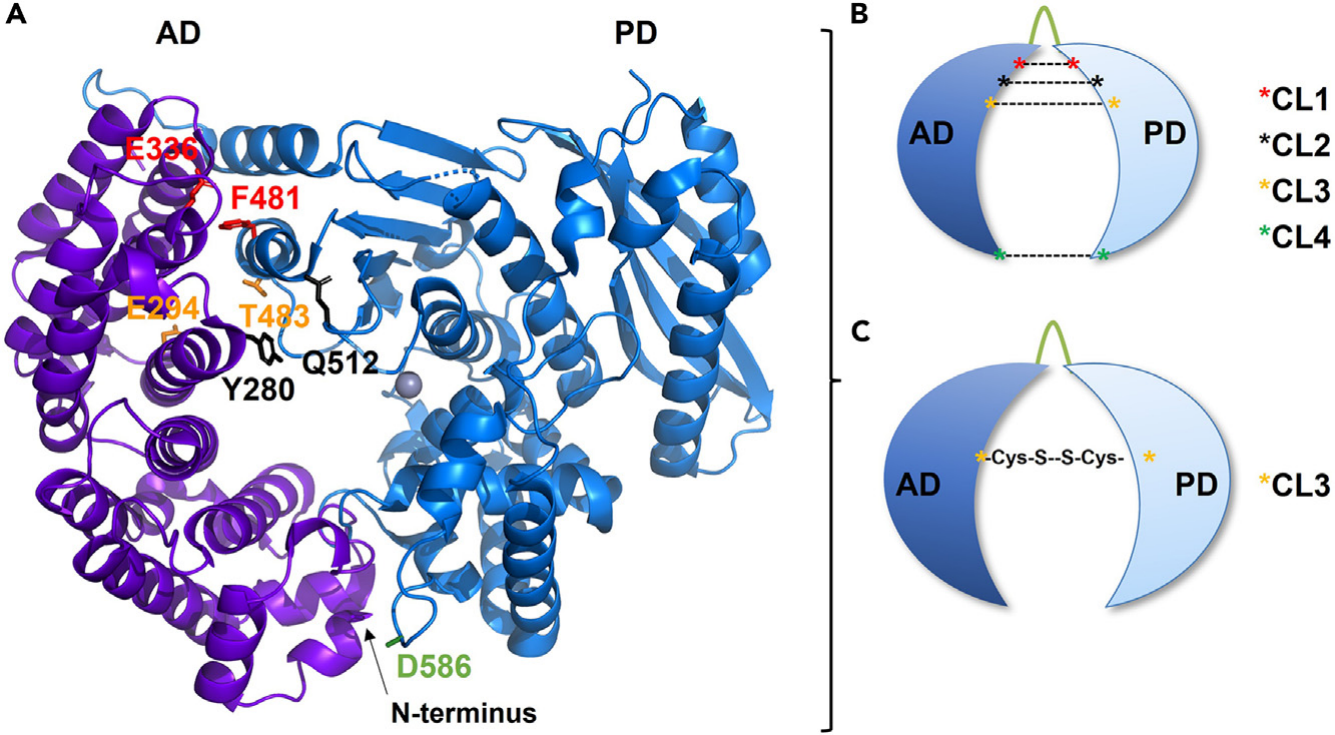
Distribution of mutation sites in ColG-CU (A) Hypothetical closed conformation of ColG-CU based on the crystal structure (PDB: 4are) with residues for the introduction of engineered cysteines highlighted in AD and PD. (B and C) Schemes illustrating the relative positions of the engineered cysteines in the mutants CL1, CL2, CL3 and CL4 (B), and, as a representative, the cross-linked variant CL3. Cross-link mutation pairs in CL1, CL2, CL3 and CL4 are E336C/F481C, Y280C/Q512C, E294C/T483C, and D586 C/C prior to N-terminal hexahistidine-tag, respectively.
  c. Based on this model, select non-conserved amino acid pairs in the AD and the PD in close proximity to each other (Figure 1A).

***Note:*** Different pairs along the vertical axis of the CU are chosen to result in variants with different degrees of inter-domain closure (*i.e.,* mutants CL1 (E336C/F481C), CL2 (Y280C/Q512C), CL3 (E294C/T483C) and CL4 (D586C/cysteine inserted before the N-terminal hexahistidine tag) (Figures 1A and 1B).

1. Design.
  a. Perform a multiple-sequence alignment of ColG and homologous proteins to identify naturally occurring cysteines (C218 and C262) and determine whether these cysteine residues are conserved or not (both non-conserved).
  b. Using the crystal structure for wild type ColG-CU (PDB: 4are), model with PyMol the closed conformation of the CU (Figure 1A).

2. Cloning.
  a. Use an expression plasmid containing the coding sequence (CDS) of the wildtype ColG-CU (WT) as template.
    ***Note:*** This expression plasmid is based on the pET-expression system encoding for an N-terminal hexahistidine tag (pET15b) to facilitate downstream purification and has an ampicillin resistance marker. The expression of the target protein can be induced by the addition of IPTG.
  b. Generate a variant of this expression plasmid with a cysteine-free CDS for ColG-CU (C218S/C262S) (CF).
  c. Confirm the removal of the native cysteines by sequencing.
  d. Using the cysteine-free expression plasmid as template, generate the expression plasmids for the four different double mutants CL1-CL4.
  e. Confirm the introduction of the engineered cysteines by sequencing.
3. Expression.
  a. Transform the mutant plasmids into an *E. coli* expression host (Nico21 DE3).
  b. Plate the transformed cells on LB-agar plates supplemented with 100 μg/mL ampicillin and incubate overnight at 37°C.
  c. Once single colonies have grown, seal the plates with parafilm and store the plates at 4°C prior to large-scale expression (maximum storage: 6 weeks).
  d. For the large-scale expression, prepare 3 L of 20 g/L LB (lysogeny broth) media for each construct.
  e. Transfer 500 mL of the media into 2.5 L baffled shake flasks, autoclave and keep the media in a sterile environment until needed.
  f. Set up for each construct 50 mL of preculture using autoclaved LB media supplemented with 100 μg/mL ampicillin.
  g. Inoculate each preculture with a single colony from the stored LB-agar plates.
  h. Incubate the preculture at 37°C with shaking at 230 rpm overnight.
  i. In the morning supplement all autoclaved flasks with 500 mL LB media with ampicillin to a final concentration of 100 μg/mL.
  j. Inoculate the media 1:100 from the preculture.
  k. Incubate the cultures at 37°C with shaking at 230 rpm until you reach an OD_600_ of 0.7–1.0.
  l. Induce protein expression by addition of 0.5 mM IPTG.
  m. Incubate at 25°C with shaking at 230 rpm overnight.
  n. Harvest the cells by centrifugation (4,000 × *g* for 10 min).
  o. Store the cell pellets at −20°C.

***Note:*** The use of Nico21 DE3 (NEB) as an expression host is encouraged, since its genotype lowers the contamination of the IMAC purified protein by endogenous *E. coli* metal-binding proteins.

***Note:*** The 3 L culture volumes were tailored for a final yield of cross-linked collagenase of approx. 10 mg.

4. Pre-purification.
  a. Suspend cell pellets with expressed protein in precooled loading buffer (2 mL/g cell wet weight).
  b. Lyse the cells on ice and purify the protein batches via IMAC using pre-equilibrated Ni-Sepharose columns by washing bound protein with 50 mL precooled wash buffer 1, wash buffer 2, and wash buffer 3 each.
  c. Elute Ni-bound protein with 250 mM imidazole using 30 mL of the precooled elution buffer.
  d. Concentrate the eluted protein using Amicon Ultra-15 devices, 10,000 MWCO (4,000 × *g*, 4°C, ×10 min).
  e. Perform size-exclusion chromatography (SEC1) at 4°C using pre-equilibrated size-exclusion chromatography column (Superdex 200 10/300 GL) with filtered and degassed size-exclusion buffer containing 10 mM ßME. Clarify the protein solution prior to loading by centrifugation (17,000 × *g* for 30 min at 4°C).
  f. Analyze the peak fractions via SDS-PAGE (12% polyacrylamide gel).
  g. Concentrate the pure monomeric SEC1 fractions using Amicon Ultra-15 devices, 10,000 MWCO (4,000 × *g*, 4°C, ×10 min) to the desired final concentration.
  h. Aliquot concentrated protein in PCR tubes.

**Pause point:** Protein samples can be flash-frozen in liquid nitrogen and stored at −80°C for several months.

### Cross-linking reaction

**Timing: 10 days at 4°C (or 3 days at 20–22°C)**

This section details the setup and time course of the cross-linking reaction.

**CRITICAL:** Select the appropriate reaction temperature for the oxidation of your target protein. For very stable proteins, the cross-linking can be performed at 20°C–22°C within 3 days. The default setting is to perform the cross-linking reaction at 4°C for 10 days to ensure the stability of your target protein throughout this preparation step.

5. Cross-linking reaction setup.
  a. Starting material per construct: ∼40 mg.
  b. Prepare 500 mL of precooled oxidation buffer in a 1 L beaker on a magnetic stirrer.
  c. Slowly add purified target protein to the oxidation buffer with gentle stirring at 4°C, until all the protein is uniformly mixed to a final concentration of 1.0 μM or lower.
  d. Add more oxidation buffer if necessary to maintain a maximum final protein concentration of 1.0 μM or lower.
  e. Add ßME to a final concentration of 1.0 mM in total.
    ***Optional:*** In case it is anticipated that the engineered cysteine sidechains might be located too distal from each other to favor spontaneous disulfide-bond formation, add a 100-fold excess of DTME dissolved in dimethyl sulfoxide (DMSO) or a similar thiol-based cross-linker to facilitate inter-domain cross-linking.
  f. Incubate the reaction for 10 days at 4°C or for 3 days at RT.
    ***Note:*** The cross-linking reaction should be maintained at a low protein concentration (1.0 μM or lower) to prevent oligomerization by intermolecular disulfide bonding, which is disfavored at low protein concentrations.
6. Protein harvest.
  a. On day 11 (4°C) or day 4 (RT) concentrate the reaction volume to 40 mL using Amicon Ultra-15 devices, 10,000 MWCO (4,000 × *g*, 4°C, ×10 min).
  b. Immediately clarify the protein by centrifugation (16,500 × *g*, 4°C, 20 min).
  c. Decant the protein solution and store protein on ice before the subsequent purification step.

**Figure 2:**
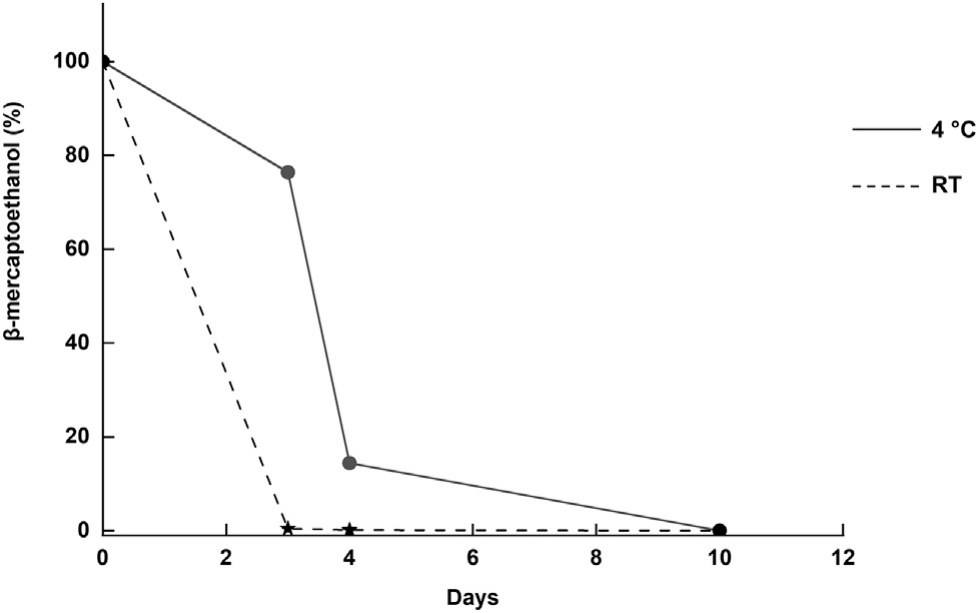
Time course of ßME evaporation at RT and 4°C in oxidation buffer The presence of the thiol-containing βME was determined using the CPM assay (Step 9). No free thiols were detected in the oxidation buffer after 3 days incubation at RT and 10 days at 4°C, respectively.

***Note:*** Optimization of cross-linking reaction was achieved by determining how long it takes for 1 mM βME to evaporate from 500 mL oxidation buffer at 4°C and RT, respectively, via the CPM assay (Figure 2). With protein in solution, no thiol detection was measured after 10 days at 4°C and after 3 days at RT, respectively.

### Isolation of monomeric cross-linked protein

**Timing: 2 days**

This section details the purification of monomeric cross-linked protein using a two-step protocol starting with Activated Thiol Sepharose (ATS) chromatography followed by gel filtration.

**Figure 3:**
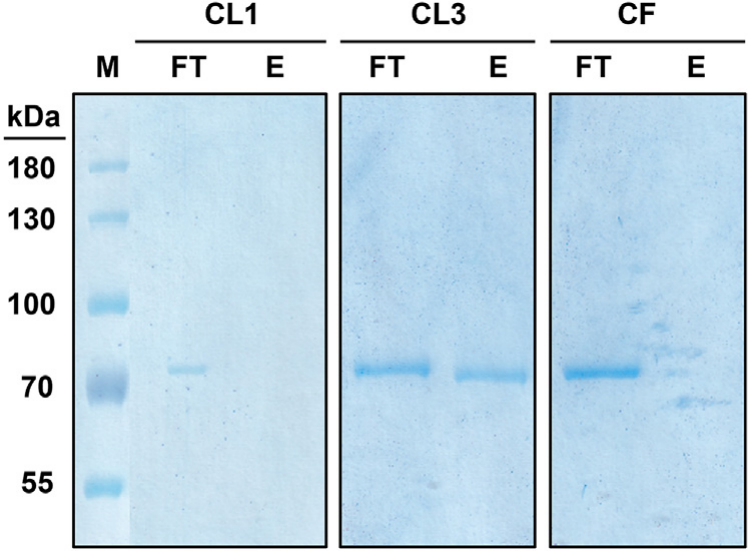
Efficiency of the oxidation procedure Non-thiol-containing species are separated from thiol-containing ones using the Activated Thiol Sepharose column. A complete oxidation of CL1 was accomplished. No protein bound to the column matrix. In the case of CL3, ∼60% of the protein formed disulfide bonds and were found in the flow-through, while ∼40% of non-cross-linked CL3 protein were captured by the column matrix and could be effectively separated from the cross-linked species obtained after elution with 20 mM βME. CF, which contains no thiols, served as a negative control. It did not bind to the column matrix and was collected entirely in the flowthrough. FT = flow-through, E = elution.

***Note:*** The binding capacity of 10 mL Activated Thiol Sepharose is approx. 30 mg of protein.

***Note:*** Because the likelihood of spontaneous disulfide formation depends on the location of the engineered cysteines relative to each other, the efficiency of the cross-linking reaction is highly construct-dependent (Figure 3). The yield of cross-linked species can be increased by the use of thiol-based cross-linkers in the cross-linking reaction.

**Figure 4:**
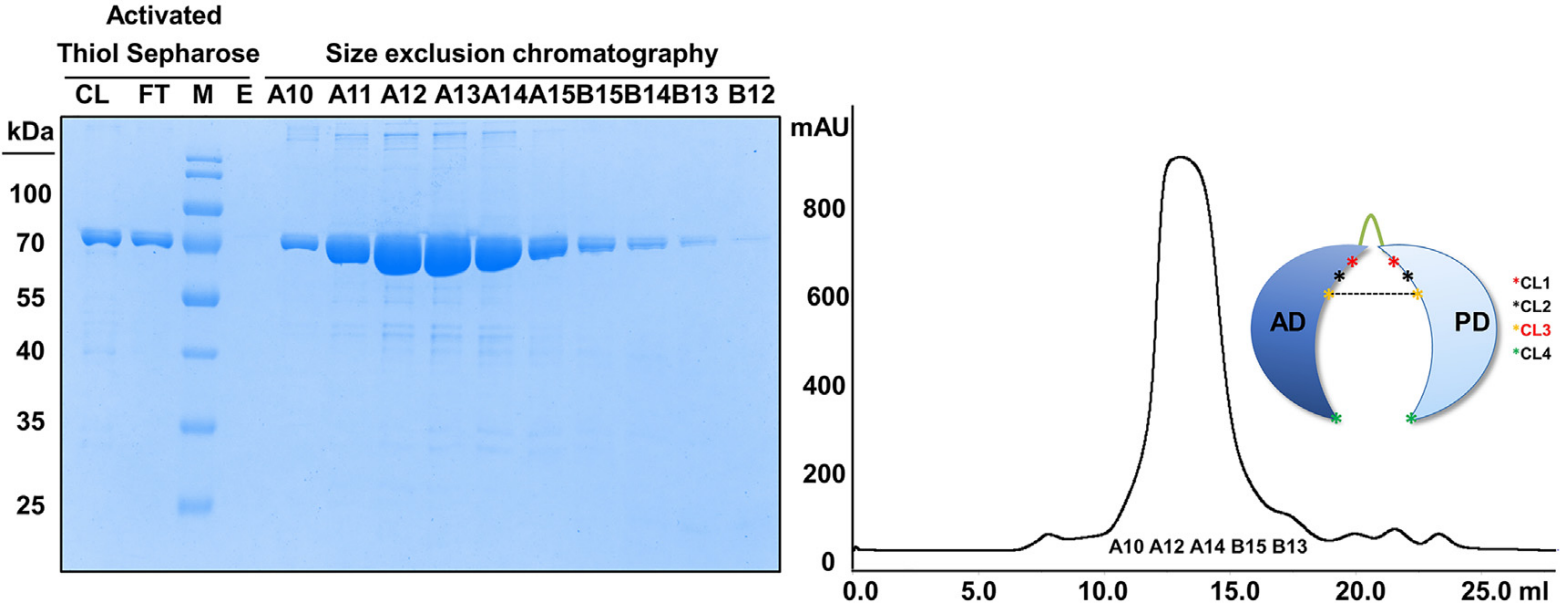
Purification of cross-linked variant CL3 by ATS and SEC The crosslinking reaction for CL3 was performed for 10 days at 4°C. In the subsequent ATS purification, the oxidized protein sample was loaded onto the Sepharose column (column load (CL)), and cross-linked protein collected in the flowthrough (FT). In the final SEC purification, referred to as SEC2, highly pure, monomeric protein was collected in the indicated peak fractions.
  i. Aliquot the protein in PCR tubes and flash freeze it with liquid nitrogen prior to long term storage at −80°C.

7. ATS chromatography.
  a. Pack 50 mL gravity flow column(s) with 10 mL of Activated Thiol Sepharose 4B resin.
  b. Equilibrate the column(s) with 100 mL of oxidation buffer.
  c. Load the column(s) with 40 mL of concentrated clarified protein.
  d. Place the column(s) on a horizontal tube roller mixer at 4°C and leave overnight to ensure proper binding of any thiol-containing molecules to the column matrix.
  e. After overnight incubation collect the cross-linked protein by gravitational flow in the flow-through and store it on ice.
  f. Wash the column with 50 mL of oxidation buffer.
  g. Obtain a 20 μL SDS-PAGE sample of the flow-through and the wash.
  h. Elute bound protein from the column:
    i. Load the column with 40 mL oxidation buffer supplemented with 20 mM βME and incubate on a tube roller mixer at RT for 5 min.
    ii. Collect the previously matrix-bound proteins in the elution via gravitational flow.
    iii. Prepare a 20 μL sample of the elution to monitor the amount of non-cross-linked protein after the cross-linking reaction.
  i. Wash the ATS column:
    i. Wash the column(s) with 250 mL 0.1%^v^/_v_ Triton™ X-100 by gravitational flow.
    ii. Wash column(s) with 500 mL of water to remove residual Triton™ X-100.
    iii. Regenerate the column(s) with 15 mL of 1.5 mM 2,2′-dipyridyl disulfide pH 8.0 and store at 4°C.
  j. Analyze the fractions of the ATS chromatography using SDS-PAGE (12%) in combination with Coomassie-R250 staining to check the efficiency of the cross-linking reaction (Figure 3).

8. Size-exclusion chromatography (SEC2).
  a. Perform the size-exclusion chromatography at 4°C.
  b. Pre-equilibrate the column (Superdex 200 10/300 GL) with filtered and degassed size-exclusion buffer containing no ßME.
  c. Concentrate the flow-through of the ATS chromatography using Amicon Ultra-15 devices, 10,000 MWCO (4,000 × *g*, 4°C, ×10 min).
  d. Clarify the concentrates by centrifugation (13,000 g, 4°C, 30 min).
  e. Load a maximum of 10 mg protein per gel filtration run.
  f. Collect all SEC2 elution fractions and obtain 20 μL SDS-PAGE samples per fraction.
  g. Analyze the fractions of SEC using SDS-PAGE (12%) in combination with Coomassie-R250 staining.
  h. Concentrate the pure monomeric SEC2 fractions using Amicon Ultra-15 devices, 10,000 MWCO (4,000 × *g*, 4°C, ×10 min) to the desired final concentration (Figure 4).

**CRITICAL:** Centrifugation of the protein sample prior to loading on the Sepharose column is paramount to avoid clogging the column with precipitated protein.

### Quality control

**Timing: 2–3 days**

The structural and functional integrity of the purified cross-linked ColG-CU variants needs to be verified prior to any downstream applications. These quality checks examine whether the final protein is fully cross-linked, properly folded, and catalytically active.

9. Verification of cross-linking by CPM assay.
  **CRITICAL:** The use of gloves, sterile equipment and materials and precise pipetting is critical for an accurate measurement. Since 7-diethylamino-3-(4-maleinimidophenyl)-4-methyl coumarin (CPM) is light sensitive, light exposure of the reagent and the plate filled with the reagent must be minimised.
  ***Note:*** CPM is only fluorescent upon conjugation with a free thiol and thus, allows the detection of free thiols in protein samples. The protocol for the assay was modified from Alexandrov et al.^18^ (Figure 5).
  ***Note:*** ColG-CU WT contains 2 cysteine residues per molecule.
  a. Prepare ColG-CU WT samples for measuring standard curves (tubes ‘Blank’ & ‘Standard 1–15’):i) Prepare 16 samples with a concentration ranging from 0–1.6 μM ColG-CU WT in 0.1 μM steps from a 10 μM stock of ColG-CU WT in a final volume of 2.0 mL using CPM buffer. ii) Store samples on ice.
  b. Prepare samples CL1-CL4:
    i. Prepare 2.0 mL samples of CL1, CL2, CL3 and CL4 at a final concentration of 1.0 μM.
    ii. Store samples on ice.
  c. Prepare CPM (1:40):
    i. Thaw 50 μL of the 4 mg/mL CPM stocks.
    ii. Add 390 μL DMSO and 1560 μL CPM buffer to generate a 1:40 working stock of CPM.
  d. Reaction setup in 96-well plate (120 μL/well):
    i. Transfer 94 μL of Blank, standards 1–15 and samples CL1-4 into 96-well plate.
    ii. Add 16 μL DMSO and 10 μL CPM.
    iii. Mix well with a multichannel pipette.
    iv. Remove bubbles using a gentle stream of ethanol vapor from a laboratory squirt bottle.
    v. Cover the wells tightly with a sealing foil and incubate at 60°C for 3 min to mildly denature the protein samples.
  e. Measure the fluorescence (E_x_: 387 nm and E_m_: 463 nm) of all wells at RT.
  f. Analysis:
    i. Subtract the blank fluorescence from all standard and sample measurements.
    ii. Generate a standard curve plotting fluorescence signal *vs.* number of thiols (μM). 1 μM ColG-CU WT corresponds to 2 μM thiols.
    iii. Interpolating from the standard curve, determine the free thiol concentration in the test samples (Table 1).
      ***Note:*** To avoid volumetric pipetting errors that would lead to inconsistent measurements, we prepare a rather large volume of 2.0 mL working stock of each protein at a final concentration of 1.0 μM.

**Table 1:** Setup of CPM assay standard curve with ColG-CU WT

Tube set up																
Tube	Blank	1	2	3	4	5	6	7	8	9	10	11	12	13	14	15
Standard (μM)	0	0.1	0.2	0.3	0.4	0.5	0.6	0.7	0.8	0.9	1.0	1.1	1.2	1.3	1.4	1.6
Thiol (μM)	0	0.2	0.4	0.6	0.8	1.0	1.2	1.4	1.6	1.8	2.0	2.2	2.4	2.6	2.8	3.2

**Figure 5:**
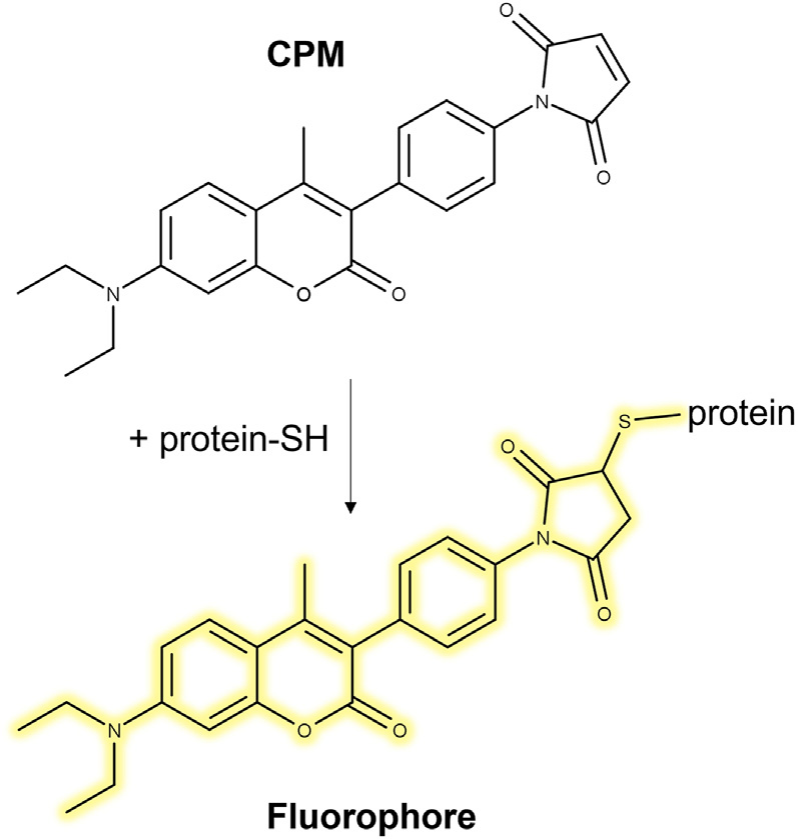
Principle of free thiol determination by the CPM assay The fluorescence of the conjugated product can be detected at 463 nm upon excitation at 387 nm.
10. Structural quality control.
  a. Collect far UV CD spectra to confirm secondary structure content:
    i. Thaw purified stocks of ColG-CU WT, CF and CL1-CL4 and store them on ice.
    ii. Set up the CD spectrometer and flush the instrument with liquid nitrogen.
    iii. Set the wavelength range for the spectral scan from 200 to 260 nm.
    iv. Set spectral bandwidth and scan time-per-point to 1 nm and 1 s, respectively.
    v. Set the temperature of measurement to RT.
    vi. If necessary, re-buffer the protein samples to remove any chloride ions.
    vii. Prepare in triplicate 200 μL at a final concentration of 5.0 μM for each protein using the CD buffer. Using 200 μL for a 0.5 mm quartz cuvette is recommended.
    viii. Collect CD spectra in triplicates.
    ix. Convert the recorded spectra data to molar ellipticity *vs.* wavelength for a direct comparison between the various protein variants (Figure 6).
      ***Note:*** The molar ellipticity is very sensitive to errors in protein concentration. Therefore, measure the concentration of all CD samples precisely.

**Figure 6:**
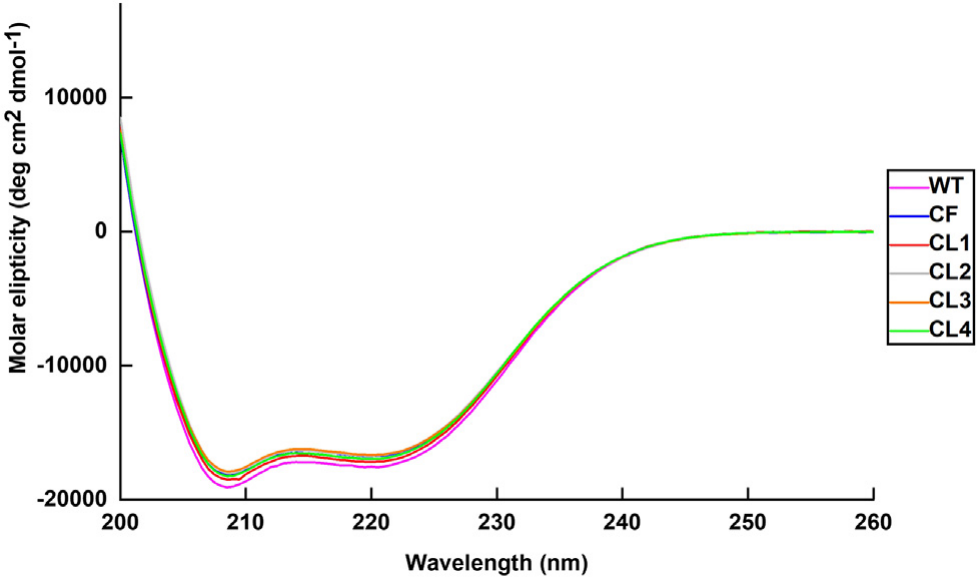
CD spectra of ColG-CU constructs The spectra show two main minima at 208 and 222 nm, indicative of a structure dominated by alpha-helices, which is in good agreement with the crystal structure of ColG-CU (PDB: 4are). The highly similar, almost identical spectra of WT and the mutants demonstrate that the cross-linking did not compromise the overall fold of the CU in the cross-linked variants.
  b. Perform differential scanning fluorimetry (DSF) experiments to assess protein stability by monitoring changes in tryptophan and tyrosine fluorescence as a function of temperature:
    i. Prepare in triplicates 100 μL of WT, CF and CL1-CL4 at a final concentration of 1.25 μM using buffer B1 and store on ice until needed.
    ii. Load the samples in triplicates into capillaries.
    iii. Record the intrinsic fluorescence at 330 nm and 350 nm, while heating the sample from 35°C to 95°C at a rate 3 °C/min.
    iv The ratio of fluorescence (350/330 nm) and the inflection temperature as a measure of thermal stability are calculated by Tycho NT. 6 software.
    v. Plot the first derivative ratio of the fluorescence ratio *vs.* temperature (Figure 7).

**Figure 7:**
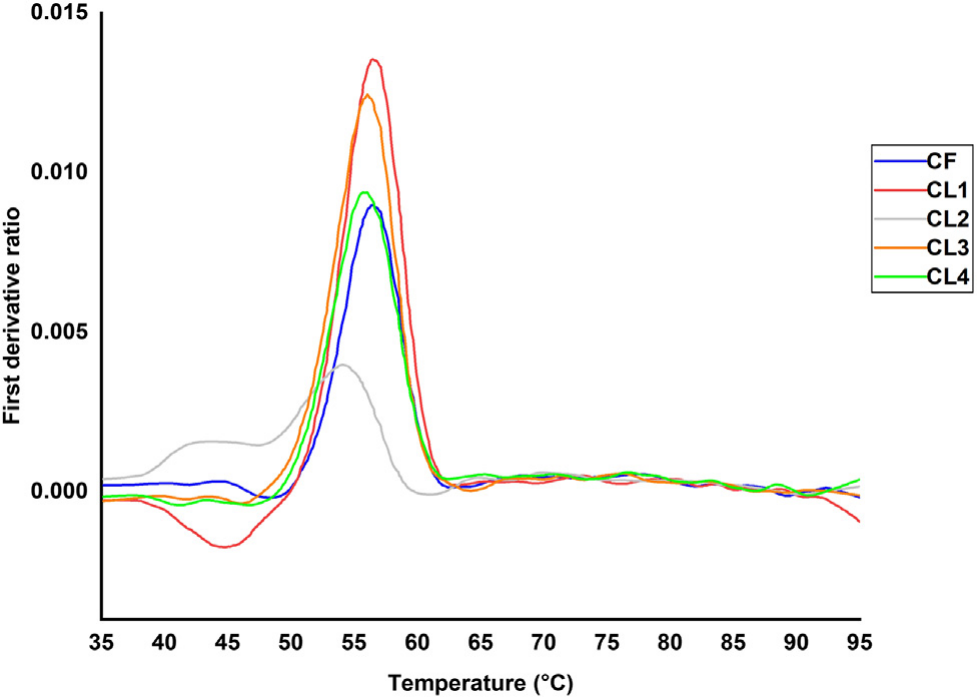
Thermal denaturation profiles of ColG mutants by DSF CL1, CL3 and CL4 exhibited comparable spectra to CF. Mutant CL2 showed significant changes that indicate a loss of structural integrity.
11. Enzymatic quality control.
  a. Prepare 500 μL with 40 nM concentration of WT, CF, and cross-linked samples CL1-CL4 using buffer B1 and store on ice.
  b. Prepare 1 mL of 10 μM quenched-fluorescent peptidic substrate in buffer B1 supplemented with 2% DMSO in an opaque tube and store at 25°C.
  c. Set up the plate reader and prepare a 96-well plate for the measurement.
  d. Prepare protein samples of CL1-CL4 in triplicate in the 96 well plate by mixing 30 μL buffer B1 and 30 μL of 40 nM cross-linked protein.
  e. Incubate at RT for 2 min and start the reactions by adding 15 μL of the 10 μM quenched-fluorescent substrate.
  f. Monitor the cleavage of the substrate for 2 min at 25°C (excitation: 328 nm, emission: 392 nm)
  g. Calculate the initial velocity (v_0_) from each reaction using linear regression of the progress curves (stay below 10% substrate conversion).
  h. Normalize the calculated enzymatic activities derived from the initial velocities to the activity of the WT (Figure 8).

**Figure 8:**
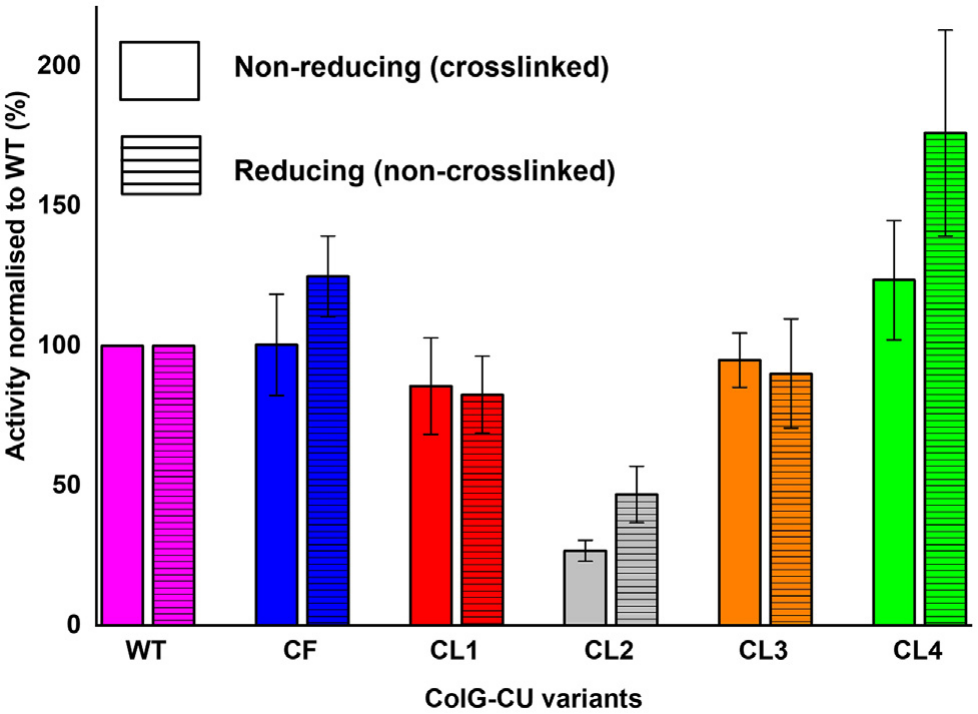
Peptide degradation assay of ColG-CU variants Compared to the WT, all mutants except CL2 displayed similar activities in the cross-linked and reduced state, confirming the structural integrity of the PD in CL1, CL3 and CL4. Data are presented as mean ± SD.

## Expected outcomes

The oxidation and isolation of cross-linked monomeric protein can be confirmed using the CPM assay. The CPM assay will reveal the presence or absence of non-cross-linked proteins in the final samples of CL1-CL4 (Table 2).

**Table 2:** Free thiol determination of ColG-CU WT and cross-linked mutants

ColG-CU collagenase	WT	CL1	CL2	CL3	CL4
Number of cysteines/molecule	2	2	2	2	2
Free thiol (μM)	2.5 ± 0.5	0.0 ± 0.5	0.3 ± 0.3	0.0 ± 0.5	0.1 ± 0.2

Results are given as mean ± standard deviation of independently purified batches.

The CD spectra and the thermal unfolding monitored by DSF are used to determine the secondary structure and stability of the protein variants. Similar CD spectra and melting temperatures compared to the non-cross-linked WT indicate that the cross-linking procedure did not compromise the overall fold and secondary structure of the cross-linked protein variants.

Finally, the peptidolytic activities of the cross-linked proteins are compared to the non-cross-linked WT. In case of successful ‘non-invasive’ cross-linking, the catalytic activities of the WT and the cross-linked variants should not differ significantly from each other.

## Limitations

This protocol puts particular emphasis on the production of highly pure, natively folded cross-linked protein. Therefore, multiple quality controls are implemented. We are aware that, in the current form, the presented approach might not be suitable for the cross-linking of proteins that contain structurally and/or functionally relevant cysteines such as cysteine proteases or disulfide-bonded proteins. For example, for proteins containing disulfide-bonds the reducing buffers would have to be complemented with an oxidized disulfide reservoir, e.g., 10 mM βME, 1 mM cystine. Such a redox system should prevent formation of artificial cysteine-modifications while preserving conformationally stabilized disulfide bonds, but would certainly require optimization.

## Troubleshooting

### Problem 1

**Figure 9:**
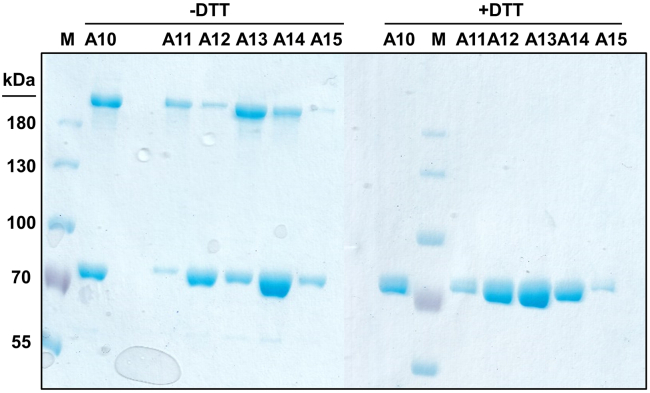
Intermolecular dimerization via disulfide linkage in CL1 The collagenase unit (79 kDa) of cross-linked proteins may form dimers (∼158 kDa) that run higher than the 180 kDa marker (-DTT), but disappear upon addition of reducing agent to the gel sample (+DTT).

Intermolecular dimerization results in oligomeric protein batches (Step 7 & 8, Figure 9).

### Potential solution

• Maintain reducing conditions (10 mM βME) in the affinity chromatography purification and first size exclusion chromatography (SEC1) runs (Step 4).
• Use lower protein concentration during the cross-linking reaction (0.1–1.0 μM) (Step 5).
• Start the cross-linking reaction with 1 mM βME in the buffer to ensure that at the start of the oxidation reaction all cysteines are completely reduced (Step 5).
• Incubate the reaction for 10 days or longer (if protein is stable at 4°C) (Step 5).

### Problem 2

Low final yield of cross-linked protein (Step 9).

### Potential solution

• Try to identify the reaction step where you lose most protein and optimize this protocol step.
• Scale up the expression volume (more than 3 L) and use larger amounts of protein for the oxidation reaction (Step 3).
• Low yields may indicate that the proposed conformational intermediate (Step 1b) is hardly populated in solution, questioning the initial assumptions.

### Problem 3

No evidence of cross-linking from the CPM measurement (Step 9).

### Potential solution

• Test the addition of a 100-fold excess of DTME or of a similar thiol-based cross-linker in the cross-linking reaction to facilitate inter-domain cross-linking (Step 5).
• Select a different pair of residues to be replaced by the engineered cysteines (Step 1c).
• No yield may indicate that the proposed conformational intermediate (Step 1b) is not populated in solution, questioning the initial assumptions.

### Problem 4

Significant deviations in the free thiol quantification are observed (Step 9).

• Standard curve preparation using molar concentrations of commercial cysteine can produce significant deviations in free thiol measurement. It may be more accurate to obtain the standard curve using a thiol-containing molecule with comparable protein background such as ColG-CU WT (Step 9).
• Free thiol measurement of cross-linked proteins using this protocol may give a negative result with the test sample. This should be interpreted as 100% cross-linking.

## Resource availability

### Lead contact

Further information and requests for resources and reagents should be directed to and will be fulfilled by the lead contact, Esther Schoenauer (esther.schoenauer@plus.ac.at).

### Materials availability

Plasmids, primers, and *E. coli* strains are available from lead contact upon request.

## Data Availability

This study did not generate any unique datasets or code.
